# Observation on a New Role for Ergotine

**Published:** 1893-04

**Authors:** T. C. Osborn

**Affiliations:** Cleburne, Texas


For Daniel's Texas Medical Journal.

### OBSERVATION ON A NEW ROLE FOR ERGOTINE.

BY T. C. OSBORN M. D., CLEBURNE, TEXAS.

TWO YEARS ago a passing wagoner stopped in Cleburne, and called upon me for medical assistance to his wife who had, he assured me, been many months afflicted with an incessant drain by monorrhagia. He had been advised, he said, by the physicians in attendance to travel by slow wagon stages,, having exhausted their favorite prescription without benefit to the patient.

I found her emaciated and pallid. The woman was young, not over 23 years of age; mother of one child, since which time the issue of blood had continued irrespective of the best assistance the husband could command.

Inspection disclosed intra-mural fibroid of considerable dimensions, and the hemorrhage interfered with further investigation, especially as it occurred at night. To control the flow, I gave her several pills of Sharp & Dohme's make, containing three grains each of ergotine, because of my confidence in the remedy, promising to visit her again next day. One pill was given every two hours in the meantime. Next day I made a closer inspection and, in addition to the enlarged posterior wall of the uterus, discovered a sub-involution, with hyperplasia of the os and the cervix of the womb.

The vagina like all the other tissues was remarkably pallid.

But as the patient expressed so much gratitude for the relief she had experienced from the pills during the night, and desired to remain under my care sometime longer, I merely commended the continuance of the pills until the next approaching menstrual period. Two weeks later, upon examination, I found that a considerable change had been made in the condition of the os and cervix uteri for the better. Her general health was also much improved; she was hopeful, whilst I was doubtful of permanence in the improvement; the remedy was, however, continued, and in addition was ordered perfect recumbent rest, a nutritious diet, and liberal draughts of generous wine several times a day.

One month later exhibited further improvement, and the treatment was continued. The issue of blood seemed to be well under control. And at the end of another month she manifested a great desire to be up from the bed, which I deprecated, but in the end consented to the change, only urging her to watch closely her condition, and go to bed again on the first approach of hemorrhage. At this time the os and cervix were improving decidedly. A month later the size of the uterus had diminished perceptibly, and at this time she begged me to permit her to go on the road again, as she was anxious to visit her mother in Ellis county; promising to keep me well informed of her improvement by letter, and to return to Cleburne on her return home for further instructions, *if she needed them*. Continue treatment.

Three months later she returned with a glow of health in her cheeks, and on examination the uterus was found still shrinking to its natural size; the hemorrhage had ceased, and I began to realize that the administration of ergotine was doing its work well. She had reduced the number of doses to one at each meal, and had perceived no ill effects from its continued administration at any time. The woman was glowing with hope of a permanent cure, whilst I was only just beginning to realize a new role for my old favorite in controlling hemorrhage in a durable sense; first, in its capacity of reducing fibroid tumors by shrinkage; and secondly, in its non-toxic effects upon the system by protracted administration.

There is a valuable lesson in this, if a more enlarged observation shall prove favorable to the hope entertained; and to prove my faith, I am now using it on every fibroid which presents itself to my attention.

Soon after my first case, I had another case of hyperlasia of the os uteri, and under a protracted use of the ergotine given similarly, the diseased condition disappeared within two months after using the remedy.

I have now under observation a polypus of the cervix uteri, in which the ergotine alone seems to be doing its work handsomely.

I have written the above with no effort at display, and I hope, without seeming redundancy, and my only wish is to call attention of the profession to the fine promise ergotine displays in this treatment of fibroid, hoping to hear further from it in the observations of other than myself.

